# Editorial: Dissemination, implementation and uptake of digital and technological interventions in practice

**DOI:** 10.3389/fdgth.2023.1349545

**Published:** 2024-01-11

**Authors:** Tom Van Daele, Christiaan Vis, Eva Van Assche, Heleen Riper

**Affiliations:** ^1^ Psychology and Technology, Centre of Expertise Care and Well-Being, Thomas More University of Applied Sciences, Antwerp, Belgium; ^2^ Centre for Technological Innovation, Mental Health and Education, Queen’s University Belfast, Belfast, United Kingdom; ^3^ Department of Public and Occupational Health, Amsterdam University Medical Centers, Amsterdam, Netherlands; ^4^ Amsterdam Public Health Research Institute, Amsterdam University Medical Centre, Amsterdam, Netherlands; ^5^ Section for Research-Based Innovation, Forhelse Research Centre for Digital Mental Health Services, Division of Psychiatry, Haukeland University Hospital, Bergen, Norway; ^6^ Section Clinical Psychology, Amsterdam Public Health Research Institute, Vrije Universiteit Amsterdam, Amsterdam, Netherlands

**Keywords:** digital and technological interventions, digital mental health, implementation, attitudes and acceptance of end-users, routine care

**Editorial on the Research Topic** Dissemination, implementation and uptake of digital and technological interventions in practice

The effectiveness of a wide range of digital interventions, like guided self-help interventions, has been well established for common mental health disorders such as depression and anxiety. In highly controlled research studies, these interventions have a substantial impact, leading to both decreases in participants' symptoms, as well as improvements in their quality of life [e.g. (1)]. Similar effects are also increasingly being reported in routine clinical practice (2). In general, guidance by mental healthcare professionals allows for sufficiently high adherence and limits drop-out (3, 4) which are key to maximise the potential of these interventions.

Research has in the past already focused on how digital interventions are both perceived, as well as received by end-users and on the effective implementation of such interventions. Although initial studies were primarily observational, i.e., describing implementation problems and barriers, research in recent years has also increasingly started to focus on developing and testing strategies to overcome these (5). However, due to the applied nature and complex settings in which these studies take place, this remains particularly challenging, especially when it comes to measuring these processes and outcomes (6). The current Research Topic focuses on this developing domain of implementation science and encompasses two topics related to digital interventions: (1) attitudes and acceptance of end-users and (2) routine care implementation.

In a first of two articles on attitudes and acceptance of end-users, Moeller et al. set up a qualitative observational study to investigate how mental health professionals decide to (not) support home-based video consultations as a part of outpatient treatment for patients with a wide range of diagnoses (i.e., anxiety disorders including obsessive-compulsive disorder, depression, bipolar disorder, schizophrenia, eating disorders, Asperger's disorder, and personality disorders). Data was obtained through field observations and informal conversations with clinicians, managers, and the implementation team in two departments of the Mental Health Service in the Region of Southern Denmark. Grounded theory was relied on as the analytical framework for subsequent analysis. This led to the identification of factors which clinicians perceived as relevant when screening and deciding on a video-consultations with patients. Important factors include the perceived added value of video-conferencing, personal and professional attitudes towards applied technologies, and technical stability and support. The article by Terhorst et al. explored to what extent a 3-min video could increase the acceptance of the general population towards smart sensing, the utilization of digital markers collected via sensors from digital devices. In a randomized controlled trial with a single post-assessment, they found that an informational video on c failed to significantly increase existing low to moderate acceptance of this particular type of technology. They nevertheless highlighted the importance for future acceptance facilitating interventions to target performance expectancy, social influence, and trust.

In an article on implementation in routine care, Van Assche et al. looked into the willingness of mental healthcare organisations to implement digital mental health as an add-on to inpatient-care, exploring both reasons for refusal as well as reasons to consider this. The Moodbuster platform for depression (7) was subsequently implemented in four organisations where professionals used Moodbuster as they saw fit in their regular therapy and patients participated on a voluntary basis. This resulted in low actual use by patients, as well as professionals. Two studies Freund et al., Tarp et al. that aimed to implement digital interventions or procedures in routine care relied on the Consolidated Framework for Implementation Research (CFIR) as a starting point to evaluate a screening procedure and a digital prevention intervention from the perspective of professionals. The CFIR framework is a possible framework which consolidates constructs from 19 implementation theories, models, and frameworks. It offers a pragmatic structure to assess implementation factors, by collecting data from individuals (to a more or lesser extent) involved in the implementation process (8). Tarp et al. assessed the implementation of a screening procedure using the itFits toolkit (9) in an iCBT routine outpatient mental health care clinic. Using cross-sectional qualitative as well as quantitative data they explored how the structured introduction of this novel process led to gradual and increasing normalization of the procedure over time. Freund et al., in turn, relied on CFIR to assess barriers to implementing digital prevention interventions (guided self-help interventions and personalised telephone coaching) for farmers with depressive symptoms. In this study, a social insurer introduced digital interventions for their insured members to complement existing prevention initiatives (e.g., group preventive services) in farmers' local vicinity. The perspectives of social insurance employees, who facilitated implementation and provide prevention services, was assessed in depth through online surveys and focus groups. Both studies demonstrate how CFIR can guide assessment of factors that influence implementation processes and might even offer inspiration for prospective use, i.e., strategy design, alongside other tools, e.g., the strategy matching tool combining CFIR and the Expert Recommendations for Implementing Change compilation (ERIC) proposed by Waltz et al. (10). At the same time, they also demonstrate the potential added value of concrete frameworks to bring further method to the complexity of routine care implementation.

In conclusion, the current Research Topic highlights the diversity of this expanding field across several exploratory and descriptive papers which each by itself help to gain more insights into further optimizing digital mental health implementation in routine care.
